# Motor Development in Children With Autism Spectrum Disorder

**DOI:** 10.3389/fped.2021.598276

**Published:** 2021-09-15

**Authors:** Ashikin Mohd Nordin, Juriza Ismail, Norazlin Kamal Nor

**Affiliations:** ^1^Faculty of Medicine, University of Cyberjaya, Cyberjaya, Malaysia; ^2^Department of Paediatrics, Faculty of Medicine, National University of Malaysia, Kuala Lumpur, Malaysia

**Keywords:** autism spectrum disorder, autism, motor delay, gross motor, fine motor

## Abstract

**Objective:** This study was conducted to determine the gross and fine motor profiles of children with autism spectrum disorder compared to typically developing children. Additionally, we also assessed if the motor delay was more pronounced with increasing age.

**Method:** This was a retrospective study involving children aged 12–60 months of age comparing motor development in children with autism spectrum disorder with typically developing children. Their developmental profile was assessed using Schedule of Growing Skills II. Descriptive statistics was used to analyse the developmental profile between the groups.

**Results:** ASD children had significant gross motor (6.7%) and fine motor delay (38.5%) compared to typically developing children, who did not show any delay. The motor delay in ASD children was more prominent in older children.

**Conclusion:** It is important to assess motor development in ASD children as there is significant motor delay in these children compared to typically developing children, and the delay becomes more prominent with age. Early detection of motor delay could allow provision of early intervention services to optimize developmental outcomes.

## Introduction

Autism spectrum disorder (ASD) is a neurodevelopmental condition that involves persistent challenges in social interaction, speech, non-verbal communication, and restrictive/repetitive behavior (1). The prevalence of ASD the United States is estimated to be 1 in every 54 children based on the data collected by the Centre of Disease Control's Autism and Developmental Disabilities Monitoring Network in 2016 (2). There are currently no reported large-scale prevalence studies in Malaysia. A feasibility study using the Modified Checklist for Autism (M-CHAT) in children aged 18–36 months performed in the Ministry of Health Malaysia (MOH) run health clinics in 2006 estimated the prevalence of ASD in Malaysia to be ~1.6 per 1,000 (3).

The diagnosis of ASD can be made clinically based on the Diagnostic and Statistical Manual of Mental Disorders, 5th Edition (DSM-5) criteria. While social communication impairment (SCI) is a one of the features, general motor impairment is not a part of the core criteria for ASD diagnosis. However, recent research has reported the presence of motor difficulty and delay in some children with ASD. Studies by using the Movement Assessment Battery for Children 2 (M- ABC2) in children aged 3–16 years reported up to 80% of children with definite overall motor skills impairment (4, 5) and a similarly, 80% of children with ASD was also reported to have gross motor delay when assessed using the Test of Gross Motor Development-2 (TGMD-2) (6). While, a more recent study examining data from the Western Australia Register, reported 35% of children aged <6 years old with an ASD diagnosis had motor difficulties based on the results of the Vineland Adaptive Behavior Scales (7). In addition to that, other earlier studies have examined the developmental trajectories of toddlers with ASD using the Mullen Scales of Early Learning (MSEL). These studies report similar observation, where by ASD children are generally delayed in all aspects of development, and this includes their gross and fine motor skills (8, 9).

However, some studies did not reach the same conclusion, for example, Ozonoff et al. in 2015 found that ASD on its own did not appear to account for motor delay in these children as delays in motor development were present in both children with ASD and children with other causes of developmental delay (DD) (10, 11).

Our study aims to shed further light on this subject especially within the context of an Asian population. The main objective of our study was to determine the profiles of gross and fine motor skills in children with ASD compared to typically developing (TD) children using the Schedule of Growing Skills II (SGS II), a developmental screening tool. We hypothesized that children with ASD were more likely to be delayed in their gross and fine motor skills compared to TD children. In addition, we wanted to assess if age was a factor in the delay of motor development by comparing motor development in younger (0–36 months) compared to older (36–60 months) ASD children. We hypothesized that children in the older age group were more likely to be detected to have delay in their motor function. In our study we did not look into more specific areas of gross and fine motor development such as walking, static and dynamic balance, praxis and hand-eye coordination.

## Methods

### Participants

This was a single-center retrospective study comparing children with ASD and typically developing children (TD). The participants were ASD children between the ages of 12 and 60 months seen at the Child Development Center (CDC) at Hospital Canselor Tuanku Muhriz (HCTM), Universiti Kebangsaan Medical Centre (UKMMC), between January 1, 2018 and June 30, 2019, and typically developing children attending the hospital for other reasons or who were children of hospital staff.

A total of 152 children were shortlisted from our database for recruitment into the study based on their age and date of clinic appointment. After reviewing the medical records, 104 were found to be eligible for analysis after excluding those with incomplete data and those who fulfilled study exclusion criteria. Exclusion criteria were: (a) Confirmed or suspected genetic syndromes or ASD with potential secondary causes, (b) Children with focal neurological findings, (c) A significant past medical history that can lead to developmental delay such as hypoxic ischaemic encephalopathy, neonatal hypoglycaemia, head injury (d) Epilepsy, (e) Significant blindness or deafness, (f) Presence of any physical disability that could impact motor development.

These were excluded to reduce other confounding factors that might affect the motor performance in these group of children. We excluded these children with recognized co-morbidities such as genetic syndromes, focal neurological findings and seizures as we postulated that brain abnormalities that may be associated with these conditions may predispose to delay in gross or fine motor development, and we wanted to determine if the diagnosis of ASD alone without these co-morbidities is associated with motor delay. This may imply a different pathway affecting neurodevelopmental progress in ASD other than brain abnormalities that present with these co-morbidities. Children with visual impairment may have motor development delay due to lack of visual input, while those with hearing impairment may be more reliant on visual cues and have differences in motor development due to this, hence the reasoning to exclude these as well.

The data of the TD children was collected by convenience sampling. A total of 74 TD children were recruited. These were children that had a follow-up appointment in the general pediatric clinic at HCTM for minor illnesses such as non-complicated asthma or mild anemia, and any healthy children that were at the general clinic waiting room, as well as children of the staff in HCTM that came voluntarily for assessment. The parents were requested to fill up a questionnaire for screening of possible concern of any developmental delay and relevant medical history. Only children with non-significant medical history and no current developmental concerns were recruited for the study.

### Instruments

#### Schedule of Growing Skills II

The Schedule of Growing Skills II (SGS II) is a developmental screening tool for children below 60 months of age. It is a tool adapted from the United Kingdom (UK) and designed by Dr. Mary Sheridan. Its purpose is to provide an accurate and reliable method of developmental screening; it is easy to use and requires little training. It is not an in-depth diagnostic tool, however it does provide pointers to the nature of the child's problem and assesses a child's development at a point of time. Although it is a British-based tool, SGS-II has been found to be a reliable and accurate tool for assessing development in disabled children in the local context based on a working paper by Haironi and Mariah from University Malaysia Sarawak in 2014 (3). In our center, the SGS II assessments are performed by trained CDC clinic nurses prior to consultation with the pediatric medical team. All children in the clinic were seen by either a pediatrician experienced in the diagnosis of ASD or pediatric medical officers under the supervision of the attending pediatrician. Children with an unclear diagnosis were referred to a special clinic with additional input from other members of the developmental team. Medical data was documented on a standardized history-taking sheet and diagnosis of ASD was made if the child fulfilled the diagnostic criteria for ASD based on the Diagnostic and Statistical Manual of Mental Disorder version 5 (DSM-5).

The SGS-II has 10 domains and is valid for use in children from birth to 60 months of age. The guideline defines “significant delay” as the developmental age being more than one age interval below the chronological age (12). This assessment uses a focused play based approach which includes clear instructions to guide the administration of the guided activities as well as gathering information from the parent or caregiver.

The diagnosis of autism spectrum disorder was made clinically based on comprehensive history, observation of child behavior and clinical examination. Diagnostic tools were used to assist clinical diagnosis, specifically criteria for ASD diagnosis under DSM 5. Early clinical diagnosis of ASD based on DSM IV, DSM 5 and clinical judgment remain at a mean age of 33 months and is stable over time in 96% of cases. Diagnostic stability is highest when clinical judgment is combined with multidisciplinary team assessment (13).

### Statistical Analysis

For statistical analysis we utilized SPSS version 20. A *p*-value of 0.5 was considered to be significant. A Fisher exact test was used to test the relationship between two categorical variables with a small sample size.

## Results

This study had a total of 178 children with 104 children from the ASD group and 74 children from the TD group. There were significantly more male children in the ASD group (88.6%) compared to the TD group and the mean age during which SGS II was administered was slightly younger in the ASD group. A higher proportion of TD children were from the Malay ethnicity compared to ASD children. Other characteristics such as parental age and maternal age at delivery did not differ between the two groups. This can be seen in Table 1.

**Table 1:** Baseline characteristics of ASD and TD children assessed.

	**ASD**	**TD**
	**Mean (SD)**	**Mean (SD)**
Age SGS II conducted	36.57 (8.75)	43.86 (10.80)
Father's age	35.47 (5.50)	35.64 (5.20)
Mother's age	33.54(4.07)	33.99 (4.21)
Mother's age at delivery	29.82 (4.94)	30.09 (4.67)
	***n*** **(%)**	***n*** **(%)**
**Gender**		
Male	92 (88.6%)	37 (50%)
Female	12 (11.4%)	37 (50%)
**Race**		
Malay	80 (77.1)	71 (95.9)
Chinese	17 (16.2)	1 (1.4)
Indian	5 (4.8)	2 (2.7)
Others	2 (1.9)	0 (0)

Our results revealed that the ASD children were significantly delayed in all domains compared to the TD children, with a large percentage (83.7%) of the ASD children having cognitive delay. A smaller percentage of the ASD children also had significant gross motor (6.7%) and fine motor delay (38.5%), whereas none of the TD children had delay in any of these domains. In our sample, a small proportion of children in the TD group had receptive and expressive language delay, although this was not statistically significant. The profile of developmental differences between the two groups is shown in Table 2.

**Table 2:** Profile of developmental delay in ASD vs. TD using SGS-II.

**Developmental domains in SGS-II**	**ASD (*n* = 104)** ***n* (%)**	**TD (*n* = 74)** ***n* (%)**	**Fisher's exact** **test (*p*-value)**
**Locomotor**			
Developmental delay	7 (6.7)	0 (0)	**0.02**
No developmental delay	97 (92.4)	74 (100)	
**Manipulative**			
Developmental delay	40(38.5)	0 (0)	**<0.01**
No developmental delay	64 (61.5)	74 (100)	
**Visual**			
Developmental delay	47 (45.2)	0 (0)	**<0.01**
No developmental delay	57 (54.8)	74 (100)	
**Hearing & language**			
Developmental delay	92 (88.5)	2 (2.7)	**<0.01**
No developmental delay	12 (11.5)	72 (97.3)	
**Speech & language**			
Developmental delay	96 (92.3)	6 (7.6)	**<0.01**
No developmental delay	8 (7.7)	68 (91.6)	
**Interactive social**			
Developmental delay	67 (64.4)	0 (0)	**<0.01**
No developmental delay	37 (35.6)	74 (100)	
**Self-care social**			
Developmental delay	59 (56.7)	0 (0)	**<0.01**
No developmental delay	45 (43.3)	74 (100)	
**Cognitive**			
Developmental delay	87(83.7)	0 (0)	**<0.01**
No developmental delay	17 (16.3)	74 (74)	

*Bolded Fisher exact test value: significant at p < 0.05*.

We also found that all the ASD children with gross motor and manipulative delay had concomitant cognitive delay.

From our study, we found that all children who screened positive for significant gross motor delay were from the older 37–60 month-old cohort. A bigger proportion of children from the older age range also had fine motor delay compared to those in the younger age range. The results are presented in Table 3.

**Table 3:** Locomotor and manipulative skills in ASD children at 0–36 and 37–60 months using SGS-II.

	**Chronological Age**		
**Developmental domain**	**0–36** **months** ***n* (%)**	**37–60** **months** ***n* (%)**	***p*-value** **(Fisher Exact** **test)**
**Locomotor**			
Developmental delay	0 (0)	7 (13.2)	**0.01**
No developmental delay	51 (100)	46 (86.8)	
**Manipulative**			
Developmental delay	12 (23.5)	28 (52.8)	**0.03**
No developmental delay	39 (76.5)	25 (47.2)	

*Bolded Fisher exact test value: significant at p < 0.05*.

As we had performed convenience sampling and noted some differences in sex and age between the ASD and TD groups, we attempted to control for these by repeating the analysis with only a proportion of ASD children who were a closer match in terms of sex and age with TD children (Results in Appendix 1). This did not affect the results, and we found that gross and fine motor delay became more prominent with increasing age in the ASD group and there were no children in the TD group that had motor delay.

## Discussion

We set out to determine the profiles of gross and fine motor skills in children with ASD compared to typically developing (TD) children in CDC UKMMC using SGS II, a developmental screening tool, and to assess if motor development delay was more pronounced in younger compared to older children with ASD. In our study we found that children with ASD had both significant gross motor delay (*n* = 7; 13.2%) and fine motor delay (*n* = 38; 52.5%), whereas delay in motor development was not seen in TD children. Significant gross motor delay in ASD children were seen only in those who were older (37–60 month old). For fine motor delay, a bigger proportion of the children from the older age range showed significant delay compared to the younger children (50.9 vs. 21.6%). In general, we found that children with ASD had significant developmental delay in all domains assessed by SGS II compared to typically developing children.

Our results are comparable to previous research published on ASD showing gross and fine motor delay in ASD children become more significant with increasing age, particularly work by Landa's group (14). It is not unexpected that a proportion of children with ASD would have global developmental delay which includes delay in the gross and fine motor domains. In our study, we were not able to ascertain if the presence of motor delay is associated with ASD purely, or if the motor delay is compounded by concurrent global development delay. It is well-established that intellectual disability is one of the known co- morbidities in ASD (15, 16). In our sample, a high proportion of ASD children were detected to have significant cognitive delay and all the children with gross motor delay were delayed cognitively. Current available literature available is not yet able to parse out the relationship between ASD and DD or intelligent quotient (IQ) scores) to gross motor delay due to the disparate nature of these studies. A recent review summarized that motor proficiency deficit can be present throughout the spectrum regardless of IQ (17). A more recent study by Kaur concluded that both fine and gross motor skills are related to the IQ level but not severity of ASD (18). In general, motor delay is present in children with ASD or DD and they are both significantly more delayed compared to their TD counterpart (10, 19).

The results of our current study are also consistent with previous studies with regards to the relationship between the age of a child with ASD and detection of motor delay, whereby motor delay becomes more prominent as the child grows older (9, 14). Even though there is evidence that motor delay could be picked up as early as 14 months in children with ASD (14), SGS II is a developmental screener and may not be as sensitive in detecting motor delay at an early age compared to more detailed and diagnostic developmental assessment tools that have been used in other studies such as the TGMD-2, MESL, M-ABC2, Bayley Scale of Infant Development II (BSID II) and Peabody Developmental Motor Scales, 2nd edition (PDMS-2) (6, 11, 14, 20). However, despite being a screening tool, SGS II was still able to pick up these differences at an older age, showing it potentially has good utility in older children for certain domains.

A previous study reported that ~33% of children with ASD have concomitant intellectual disability with IQ less that 70 (21). In our study we found that a large portion of children were also cognitively delayed, which may be associated with concurrent gross and fine motor delay. Unfortunately, we were not able to test this hypothesis with our study instrument. Praxis error may also give a clearer relationship between motor development and the severity of ASD (18). Unfortunately, we were not able to ascertain praxis error in our study due to the instruments we used.

From the review of literature, current research has not been able to conclusively use motor delay as an early marker for diagnosis of autism due to the disparity of research results (22). However, identifying motor delay may aid therapists in provision of more personalized therapy as motor skills may be linked to the development of other domains. LeBarton and Landa reported that early motor skills delay may be associated with delay in language development (23). In addition, gross and fine motor skills are also important to assist in advancement of adaptive living (24) and socialization skills (25–27). Understanding that motor domains may be affected in children with ASD will provide clinicians and practitioners working with ASD children better awareness of the spectrum of difficulties these children face and enable earlier detection and intervention. These in turn are likely to improve outcome and quality of life of these affected children.

Our research found that about 8% of the children from the TD group that were screened had language delays despite their parents not reporting them as having any developmental concerns on initial questioning. While none of these children with language delays had features to suggest ASD, this finding is important in highlighting the hidden problem of developmental delay in children, specifically language delay, of which parents may not be aware. The language delay was picked up by the SGS II screening assessment which involves both; the language used by the child during assessment as well as focused questions on the child's language development in the parent interview as recommended by the assessment. The prevalence of language delay in our sample is slightly higher than expected and may not reflect community prevalence. The general prevalence of language delay is reported to be between 2.6 and 6% (28, 29). Many of the TD children were the children of our hospital staff that came voluntarily for assessment. We postulate that some parents may have come for screening to confirm or alleviate their unexpressed concerns regarding their child's development, although they did not state any concern regarding development specifically in the screening questionnaire.

There were a few limitations in our study. Firstly, we used the SGS II, which is a developmental screening tool instead of a diagnostic tool. The use of a screening tool may affect the accuracy of the developmental profile obtained, with subtle differences such as balance and coordination skills potentially not detected. In addition, we did not collect data regarding comorbidities such as intellectual disability or developmental disability, which may have been the main driver for the differences of motor development seen in ASD children compared to TD children. A systematic review by Paquet has suggested that a more complete assessment of motor function (walking, static and dynamic balance, coordination, manual praxis, hand-eye coordination) using standardized assessment tools taking into account the neuro-cognitive level would enable us to better understand the relationship between motor development and ASD (19). Thus, in future studies, use of more specific developmental tools and instruments for assessments of co-morbidities could further improve the study design and parse out true associations. In addition, in younger children, a more sensitive tool could be considered to assess motor delay, as we found the SGS II did not detect differences in younger ASD children.

Apart from this, we also found that there was a higher number of male children within the ASD group, with a ratio of 2.5 males: 1 female child. The children in the ASD group were also slightly younger. The excess in male children in the ASD group is not surprising as it has been well-established that there is a male preponderance in ASD, with current literature citing a male to female ratio between 3:1 and 4:1 (30, 31). These differences may be significant as recent evidence suggest that fundamental motor skills in boys and girls may defer across the preschool period (32) and it has also been reported that 4-year-old boys assessed by the M-ABC were found to score lower on the manual dexterity and balance score compared to the girls (33). We attempted to address this by re-analyzing the data and matching ASD and TD children by gender and age and found that the results were not altered significantly. In addition, our study did not investigate other compounding factors that may affect motor development such as the impact of growth, diet and environmental stimulation that may influence motor development.

In conclusion, our study shows that children with ASD are more likely to have delay in both gross and fine motor domains compared to TD children, and that this delay is more pronounced in older children with ASD. Even though SGS II is a screening tool, it may be useful in a resource-limited setting to assess developmental profile children with ASD. Earlier detection of developmental delay is imperative to enable provision of early intervention to optimize long term outcomes. In children with ASD, early intervention services should also promote gross and fine motor development in addition to communication and behavioral intervention. These motor skills are essential for overall developmental outcomes for all children.

## Data Availability Statement

All datasets presented in this study are included in the article/supplementary material.

## Ethics Statement

The studies involving human participants were reviewed and approved by the Universiti Kebangsaan Malaysia institutional research and ethics committee (Project code: JEP- 2019-302). Written informed consent to participate in this study was provided by the participants' legal guardian/next of kin.

## Author Contributions

All authors listed have made a substantial, direct and intellectual contribution to the work, and approved it for publication.

## Conflict of Interest

The authors declare that the research was conducted in the absence of any commercial or financial relationships that could be construed as a potential conflict of interest.

## Publisher's Note

All claims expressed in this article are solely those of the authors and do not necessarily represent those of their affiliated organizations, or those of the publisher, the editors and the reviewers. Any product that may be evaluated in this article, or claim that may be made by its manufacturer, is not guaranteed or endorsed by the publisher.

## Appendix 1

**Table:** Profile of developmental differences in ASD vs. TD matched for age using SGS-II.

	**ASD (*n* = 55)** ***n* (%)**	**TD (*n* = 56)** ***n* (%)**	**Fisher's exact** **test (*p*-value)**
**Locomotor**			
Significant difference	4 (7.3)	0 (0)	0.057
No significant difference	51 (92.7)	56 (100)	
**Manipulative**			
Significant difference	21 (38.2)	0 (0)	**<0.01**
No significant difference	34 (61.8)	56 (100)	
**Visual**			
Significant difference	22(40)	0 (0)	**<0.01**
No significant difference	33 (56)	56 (100)	
**Hearing & language**			
Significant difference	46 (83.6)	2 (3.6)	**<0.01**
No significant difference	9 (16.4)	54 (96.4)	
**Speech & language**			
Significant difference	47(85.5)	3 (5.4)	**<0.01**
No significant difference	8 (14.5)	53 (94.6)	
**Interactive social**			
Significant difference	35 (63.6)	0 (0)	**<0.01**
No significant difference	20 (36.4)	56 (100)	
**Self-care social**			
Significant difference	29 (52.7)	0 (0)	**<0.01**
No significant difference	26 (47.3)	56 (100)	
**Cognitive**			
Significant difference	46 (83.6)	0 (0)	**<0.01**
No significant difference	9 (16.4)	56 (74)	

*Bolded Fisher exact test value: significant at p < 0.05*.
